# Economic assessment of traditional surgical intervention versus use of a new innovative radiofrequency based surgical system in device replacements

**DOI:** 10.1371/journal.pone.0192587

**Published:** 2018-03-06

**Authors:** Alexander Kypta, Hermann Blessberger, Juergen Kammler, Alexander Nahler, Kurt Neeser, Michael Lichtenauer, Christoph Edlinger, Joerg Kellermair, Daniel Kiblboeck, Thomas Lambert, Johannes Auer, Clemens Steinwender

**Affiliations:** 1 Department of Cardiology, Kepler University Hospital, Linz, Austria; 2 Department of Cardiology, Paracelsus Medical University of Salzburg, Salzburg, Austria; 3 Analytica Laser International Inc., Lörrach, Germany; 4 Department of Internal Medicine I, St. Josef Hospital, Braunau, Austria; Klinikum Region Hannover GmbH, GERMANY

## Abstract

**Introduction:**

Intra-operative complications like mechanical damages to the leads, infections and hematomas during generator replacements of implantable pacemakers and defibrillators contribute to additional costs for hospitals. The aim of this study was to evaluate operation room use, costs and budget impact of generator replacements using either a traditional surgical intervention (TSI) with scissors, scalpel and electrocautery vs. a new radiofrequency energy based surgical system, called PEAK PlasmaBlade^TM^ (PPB).

**Materials and methods:**

We conducted a retrospective analysis of a population including 508 patients with TSI and 254 patients with PPB who underwent generator replacement at the Kepler University Hospital in Linz or the St. Josef Hospital in Braunau, Austria. The economic analysis included costs of resources used for intra-operative complications (lead damages) and of procedure time for TSI vs. PPB.

**Results:**

Proportion of males, mean age and type of generator replaced were similar between the two groups. Lead damages occurred significantly more frequent with TSI than with PPB (5.3% and 0.4%; p< 0.001) and the procedure time was significantly longer with TSI than with PPB (47.9±24.9 and 34.1±18.1 minutes; p<0.001). Shorter procedure time and a lower rate of lead damages with PPB resulted in per patient cost savings of €81. Based on estimated 2,700 patients annually undergoing generator replacement in Austria, the use of PPB may translate into cost savings of €219,600 and 621 saved operating facility hours.

**Conclusion:**

PPB has the potential to minimize the risk of lead damage with more efficient utilization of the operating room. Along with cost savings and improved quality of care, hospitals may use the saved operating room hours to increase the number of daily surgeries.

## Introduction

Due to the aging population, the number of patients eligible for the implantation of a pacemaker (PM), a cardiac resynchronization therapy pacemaker (CRT-P), a CRT pacemaker with defibrillation therapy (CRT-D) or an implantable cardioverter-defibrillator (ICD) device is rising every year [1–3]. All such devices are placed in the chest just beneath the skin to help control the abnormal heart rhythms or improve the pumping function of the heart. The main component of an implantable pacemaker is the generator. The generator consists of a battery, the impulse generator itself as well as control electronics. Electrodes connect the generator to the heart tissue. They usually travel in large body veins from the generator pocket to the right ventricle or the right atrium. Electrodes are capable of both sensing the heart’s own electrical activtiy and delivering low energy pulses to the heart muscle (pacing). Pacemakers are used to treat various types of bradyarrhythmias such as sick sinus syndrome or complete heart block. Depending on the exact type of arrhythmia, pacemakers with either one or two leads are implanted, whereas pacemaker systems with three leads are used in heart failure patients to achieve resynchronization between the right and the left ventricle (cardiac resynchronization therapy, CRT) that is often lost in heart failure [4]. Synchronized contraction of both ventricles improves cardiac output and performance. Besides leads in the right atrium and ventricle, the third lead is placed in the coronary sinus to pace the left ventricle. Similar to pacemakers, an implantable cardiac defibrillator/cardioverter (ICD) also consists of a battery, a computerized generator, and wires with sensors at their tips, monitoring and stimulating the heart's electrical activity. However, an ICD is also capable of delivering high energy electrical shocks or fast bursts of pacing to the myocardium that can terminate life threatening tachyarrhythmias by rebooting the heart’s conduction system. Ventricular tachycardias or fibrillation are such arrhythmias that can cause a critical reduction in cardiac output that leads to a shortage of vital organ perfusion. Thus, an ICD is used for patients deemed at high risk for such life threatening tachyarrhythmias like sudden cardiac death survivors, patients with advanced stages of heart failure or patients diagnosed with certain electrical heart diseases (channelopathies) [5]. Each ICD can also work as a pacemaker. Furthermore, an ICD and CRT system can also be combined (CRT-D system).

All of these devices need to be checked regularly as their proper function can be impaired due to various reasons. One of the most common reason is the depletion of batteries. Pacemaker batteries last between 5 and 15 years (8 to 10 years on average), depending on how active the pacemaker is. With the growing number of pacemaker, ICD and CRT implantations, the demand for generator replacements due to battery depletion and functional issues is increasing. Patients who undergo a surgical replacement are at risk of clinical complications such as infections and mechanical damage to the lead(s). These are mostly caused by the use of scissors or scalpels during blunt dissection of the generator from the surrounding fibrotic capsule, especially if leads are located above or below the generator. Routinely, electrocautery is used during cardiac device implantation and generator replacements for both local hemostasis and tissue dissection. Since the material covering the leads has a low thermal stability, the use of direct cautery may cause severe damage to the leads resulting in their malfunctioning. Due to this fact, stripping off fibrous tissue from leads with scissors and scalpels may result in additional costs for the new leads, longer surgery time as well as longer operating room use.

The PEAK PlasmaBladeTM is a surgical device that uses brief and high frequency energy pulses to dissect the tissue. Due to this specific technology, the PEAK PlasmaBladeTM works at significantly lower temperatures than traditional electrocautery (40–170°C vs. 200–350°C). Thus, the PEAK PlasmaBladeTM may have several advantages over the standard approach with surgical scissors and conventional electrocautery. Preciser incisions and less tissue damage with a substantially lower risk of lead damage may both contribute to increase efficiency of the procedure.

The aim of the underlying analysis was to assess the operation room use, costs and budget impact of the currently applied traditional surgical intervention (TSI) with the use of scissors, scalpels and electrocautery compared to the innovative surgical system, called PEAK PlasmaBladeTM (PPB) in the Austrian healthcare setting.

## Materials and methods

All procedures performed were in accordance with the ethical standards of the instutional and national research committee and with the 1964 Declaration of Helsinki, its later amendments or comparable ethical standards. Ethical approval was granted by the ethics committee of the province of Upper Austria. The patient sample considered in the present analysis is based on two patient registries in Austria (Kepler University Hospital Linz and St. Josef Hospital Braunau). In total 818 patients (Linz n = 763; Braunau n = 55) undergoing an elective surgical generator replacement of a pacemaker (PM), a cardiac resynchronization therapy pacemaker (CRT-P), a cardiac resynchronization therapy pacemaker with defibrillator (CRT-D) or an implantable cardioverter-defibrillator (ICD) device between 2003 and 2015 were included. This dual center patient cohort was evaluated in order to confirm the robustness and applicability of findings assessed by a previous single center study [6]. All generator replacements were performed by three experienced operators with two of them having more than 20 years practical working experience and one of them having more than 7 years. The following parameters were analyzed: gender, age, type of surgical method (traditional surgical intervention (TSI) using scissors, scalpels and electrocautery; new surgical system, called PEAK PlasmaBladeTM (PPB) [Medtronic Inc.; USA]), type of replaced devices, the duration of surgical interventions, complication rates and duration of postoperative hospital stay. The implanted generators were provided by different manufacturers such as Medtronic Inc., Vitatron, St. Jude Medical, Boston Scientific, Sorin and Biotronik. A lead damage was defined as either an insulation defect upon visual inspection of the lead or an impairment of electrical parameters during generator replacement or during the index hospital stay. In the case of a lead damage, a new lead was implanted within the same procedure and the old one was either left in place or extracted.

### Statistical analyses

A non-parametric two-sided Wilcoxon test was used for the analysis of procedure time since data were not normally distributed. A two-sided Fisher’s exact test was applied for the binary complication endpoints. A significance level of 5% was chosen for each test. Due to the retrospective and exploratory nature of the analysis, an adjustment for multiple testing was not considered. Statistical analyses were performed for demographic data, procedure time (defined as time from first skin incision until end of surgery), lead damage during device replacement, revision for hematoma, revision for infection, hematoma with conservative treatment, occurrence of any complication (defined as any lead damage, revision for hematoma, revision for infection or hematoma with conservative treatment), and occurrence of any major complication (defined as lead damage, revision for hematoma, revision for infection). In order to eliminate the effect of treatment-selection bias, the propensity score matching was performed in study population. The propensity scores (PS) were estimated using logistic regression model accounting for age and gender. Every PEAK PlasmaBlade^TM^ patient was matched with two electrocautery patients with a matching error of PS set at 0.05. The balance was tested for statistical difference on age and gender between two arms in matched population. Statistical analysis was performed using SAS 9.3 (SAS Institute, Cary, NC).

### Cost assessment and budget impact analysis

The cost assessment is based upon the direct costs associated with TSI and PPB, which included the cost of consumables for electrocautery and PEAK PlasmaBlade^TM^, the costs due to intra-operative complications and the duration of operation time. The complication related cost comprised the cost of re-implanted leads, additional X-ray, additional hospital stay and physician visits following three months post-operation. The duration of operation for TSI and PPB has been multiplied with a weighted charge per minute for operation room use and personnel costs. For the assessment of budget impact, the overall cost difference between TSI and PPB was multiplied with the number of annually performed generator replacements in Austria.

## Results

### Study population, hospital stay and complications

**Fig 1:**
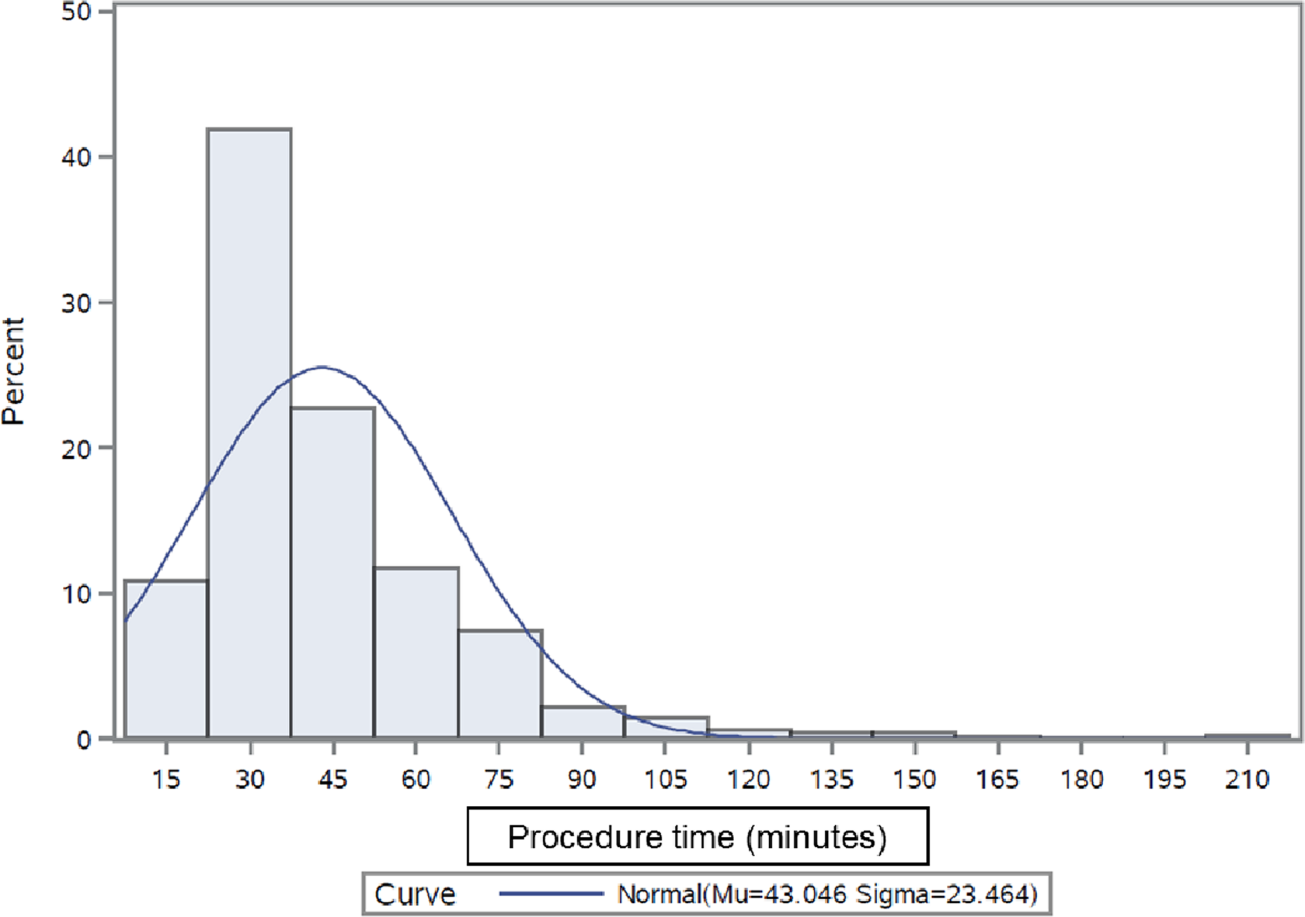
Distribution of procedure time (all patients).

The original cohort from the two hospitals in Austria included n = 818 patients. Due to a significant imbalance of the mean age between the PPB (mean 76.5 ±12.4 years) and TSI (74.5±12.5 years) group, these two groups were adjusted by propensity score matching resulting in n = 762 patients. The overall TSI group comprised 508 patients while PPB group consisted of 254 patients. The two groups were not significantly different with respect to mean age (TSI = 74.3±12.3; PPB = 75.7±12.7 years; p = 0.113) and proportion of males (TSI = 59.8%; PPB = 60.2%; p = 0.938) (Table 1). Details about different types of cardiac devices for the two groups are enlisted in Table 2. At least one major complication (i.e. lead damage, hematoma or infection requiring evacuation) occurred in 35 patients (6.9%) of TSI and in six patients (2.4%) of PPB group (p = 0.01, see Table 3). Damages to leads were significantly higher in TSI (27 events; 5.3%: 24 PM leads and 3 ICD leads) than in PPB patients (1 event; 0.4%; p<0.001). The rate of any complication (46 events; 9.1% and 11 events; 4.3%; p = 0.019) was significantly higher in the TSI group compared with the PPB group. The most frequent complications were mild hematomas (11 in TSI group (2.2%) and 5 in PPB group (2.0%); p = 1.000) persisting for more than 7 days with no need of further treatment. No peri-procedural deaths occurred in either group. All scheduled follow-up appointments were completed by 100% of patients from both groups. The operation time (47.9 ± 24.9 minutes and 34.1 ± 18.1 minutes respectively; p<0.001, see Fig 1) and the length of hospital stay (3.2±2.7 days and 2.4±3.4 days respectively; p<0.001) were significantly longer in TSI than that in PPB (Table 4). Since the duration of hospital stay was also influenced by the changes in the local health policy over the last decade and does not necessarily represent the benefit solely attributable to PPB, this observation was not used in the current economic analysis.

**Table 1:** Demographics between electro-surgery and PEAK PlasmaBlade^TM^ groups (matched groups).

	Electro-surgery/scissors	PEAK PlasmaBlade^TM^	p-value *
Age			0.113
n	508	254	
Mean	74.3	75.7	
Standard Deviation	12.3	12.7	
Median	76.6	76.4	
Minimum	5.8	19.0	
Maximum	100.0	98.4	
Gender			0.938
Female	204 (40.2%)	101 (39.8%)	
Male	304 (59.8%)	153 (60.2%)	

* p-value is for testing the null hypothesis that population values (means or proportions) are equal among surgery groups. All tests are two-sided.

**Table 2:** Generator type distribution between electro-surgery and PEAK PlasmaBlade^TM^ groups (matched groups).

	Electro-surgery/scissors	PEAK PlasmaBlade^TM^	p-value §
n	508	254	0.002
Different generator types			
CRT-P	39 (7.7%)	32 (12.6%)	
CRT-D	16 (3.2%)	10 (3.9%)	
DDD	264 (52.0%)	137 (53.9%)	
ICD	80 (15.8%)	41 (16.1%)	
VVI	109 (21.5%)	31 (12.2%)	
Not assigned	0 (0.0%)	3 (1.2%)	

^§^Two-sided Fisher’s exact test.

DDD: dual chamber ventricular pacemaker, ICD: Implantable cardioverter-defibrillator, VVI: Single chamber ventricular pacemaker, CRT-P: cardiac resynchronization therapy pacemaker, CRT-D: cardiac resynchronization therapy defibrillator.

**Table 3:** Results for differences in complications between electro-surgery (n = 508) and PEAK PlasmaBlade^TM^ (n = 254).

Endpoint	Electro-surgery/scissors		PEAK PlasmaBlade^TM^		p-value ^§^
	n	%	n	%	
Lead damage	27	5.3%	1	0.4%	< .001*
Revision for haematoma	7	1.4%	3	1.2%	1
Revision for infection	3	0.6%	2	0.8%	1
Haematoma—conservative treatment	11	2.2%	5	2.0%	1
Summary variables					
Any Major complication	35	6.9%	6	2.4%	0.010*
Any complication	46	9.1%	11	4.3%	0.019*

^§^ Two-sided Fisher’s exact test

* indicates statistical significance at 0.05 level.

**Table 4:** Results of hospital stay and procedure time between electro-surgery and PEAK PlasmaBlade^TM^.

Endpoint	Type of surgical intervention	n	Mean	SD	Median	Min	Max	p-value §
Hospital stay	Electro-surgery/scissors	508	3.2	2.7	2.0	1	27	<0.001*
	PEAK PlasmaBlade^TM^	254	2.4	3.4	2.0	1	33	
Procedure time	Electro-surgery/scissors	508	47.9	24.9	40.0	15	205	<0.001*
	PEAK PlasmaBlade^TM^	254	34.1	18.1	30.0	15	216	

§ 2-sided Wilcoxon test

* indicates statistical significance at 0.05 level.

### Costs of complication and resource utilization

The unit cost of operation room including the personnel cost is estimated to be €7.65 per minute. Since the average duration of procedure is 47.9 minutes and 34.1 minutes for TSI and PPB group respectively, the per patient cost is estimated to be €366 and €261, resulting in per patient cost savings of approximately €105 with the use of PPB (Tables 5 and 6). Due to a significantly higher rate of lead damage in TSI than in PPB during the procedure, the lower need for lead replacements results in a cost saving of €103 per patient operated with PPB than with TSI. On the other hand, the costs for consumables are €127 higher for PPB than for TSI which is primarily driven by the higher cost for a single-use tip of PEAK PlasmaBlade^TM^ in comparison to that for the cautery tip. The use of PEAK PlasmaBlade^TM^ instead of scissor/conventional electrocautery resulted in overall per patient cost saving of €81. In 2014, according to the EHRA White Book, about 2,700 implanted PM and ICD generators were replaced in Austria. Use of PEAK PlasmaBlade^TM^ instead of the traditional approach would result in an annual cost saving of about €219,600 and 621 saved operating room hours for this indication alone.

**Table 5:** Cost components used for the economic analysis for personnel and facility (personal communication Kepler University Hospital Linz and St. Josef Hospital Braunau).

Personnel costs	Per minute rate (€)
• Physician	2.54
• Nurse	1.12
• Assisting Nurse	0.82
• Medical technician	1.18
• Assistant	0.80
Sub-total	6.46
Facility costs	Per minute rate (€)
• Cath-lab	1.19
	Per minute rate (€)
Total cost per minute	7.65

**Table 6:** Per patient cost analysis.

		Electro-surgery/scissors		PEAK PlasmaBlade^TM^	
Complications	Cost per replaced lead (€)	Percentage of patients	Cost per patient (€)	Percentage of patients	Cost per patient (€)
Lead damage (ICD)	1790	0.59%	10.53	0.0%	0.00
Lead damage (PM)	465	4.72%	21.93	0.4%	1.86
X-ray following lead replacement	50	5.3%	2.65	0.4%	0.20
2 additional days hospital stay following lead replacement	672	5.3%	71.26	0.4%	5.37
Punction Set	22	5.3%	1.17	0.4%	0.09
Additional physician visit (3-month post op)	56	5.3%	2.97	0.4%	0.22
Total cost of damaged lead replacement		110.52		7.74	
Resource use	Cost per minute operation room use (€)	Minutes per patient	Cost per patient (€)	Minutes per patient	Cost per patient (€)
procedure cost (cath lab time and personnel cost)	7.65	47.9	366.44	34.1	260.87
Consumables (cauter or PEAK-tip)	—	—	40	—	167
Total cost of resource use (€)		406.44		427.87	
Overall cost (sum of damaged lead replacement and resource use costs)		516.95		435.61	
Cost saving with PEAK PlasmaBlade^TM^ (€)	81.34				

### Subgroup analysis

A subgroup analysis has been performed to assess the outcomes for the Kepler University Hospital in Linz and the St. Josef Hospital in Braunau separately:

If only patients treated at the Kepler University Hospital in Linz were evaluated (TSI group: 474 patients; PPB group had 237 patients), the comparison of PPB vs. TSI based on the matched patients indicated a benefit in favor of PPB regarding the duration of procedure (minus 15.2 minutes, p<0.001), proportion of lead damages (minus 4.7%, p<0.001), major complications (minus 3.6%, p = 0.042) and cost savings of €83, which is similar to the findings of the overall matched group in the main analysis. The differences between TSI (n = 24) and PPB (n = 31) derived from the patient cohort of the St. Josef Hospital in Braunau indicated a non-significant trend of an improved outcome with the use of PPB with respect to the duration of procedure (minus 3.7 minutes), proportion of lead damages (minus 4.2%) and major complications (minus 4.2%). The cost saving of using PPB instead of TSI (€ 17) was considerably lower than in the main analysis and for Linz.

### Sensitivity analysis

The unmatched cohort derived from the registries of the two hospitals in Austria comprised n = 818 patients. After matching by age and gender the analyzed population included n = 762 patients with a generator replacement in these hospitals. To test the impact of matching the population, the statistical analysis and cost comparison was also performed for the unmatched population. This analysis indicated that the differences between PPB and TSI regarding duration of procedure (minus 14.1 minutes, p<0.001), proportion of lead damages (minus 5.2%, p<0.001) and major complications (minus 5%, p = 0.003), as well as the cost saving (€ 89) are similar to the findings in the matched population.

## Discussion

Despite being a routine intervention in everyday clinical practice, generator replacements are associated with a moderate complication risk that increases with the number of leads and is higher for ICD compared with pacemaker replacements [7–9]. In the large prospective REPLACE registry comprising about 1,700 patients the overall number of major complications within 6 months after generator replacement ranged between 4.0% and 15.3%. Lead dislodgements, lead malfunctions and lead-interface problems accounted for 1.1% up to 8.4% of complications depending on the subgroup [7]. Due to the demographic shift, the number of pacemaker and ICD implantations as well as consecutive generator replacements will rise in the future. It was reported for Austria that 8,272 PM, 1,362 ICDs, 420 CRT-P and 816 CRT-D were implanted in 2014, representing an increase of implanted devices of about 4.5% compared to 2013. Despite regional differences in Europe the number of PM, ICD and CRTs implantations ha been increasing over the past years, especially in northern countries [10]. Encapsulation of devices with fibrotic tissue over time makes careful dissection of the leads necessary before generator replacements [11–12]. With the number of generator replacements on the rise, lead damages that necessitate unplanned excess implantations of new leads will highly likely burden health care budgets. Our investigation proved that the risk of lead damages can virtually be eradicated by the use of the PEAK PlasmaBlade^TM^ which translates into cost savings. The significantly shortened procedure time further helps to utilize available resources as effective and efficient as possible. This seems especially interesting for hospital managers who are often facing cost containment policies in the healthcare sector in industrialized countries nowadays. Due to its design and technical specifications, PEAK PlasmaBlade^TM^ is ideal for device change. Although, the PEAK PlasmaBlade^TM^ showed a significantly lower incidence of lead damages (PPB 0.4% vs. TSI 5.3%), the complication rates in terms of infections and conservative treatment of hematomas were not significantly different between the two surgical approaches. It has to be noted that much less data was available for the new PEAK PlasmaBlade^TM^ than for the group operated upon using the conventional surgical approach. As opposed to the data from the prospective REPLACE registry presented above, a recent retrospective analysis of a database comprising more than 45,000 patients with generator replacements between 2009 and 2013 revealed a lower rate of lead damages. The incidence was between 0.5% and 2.0% with a higher rate in CRT-D than in pacemaker carriers [13]. In contrast to these data, the observed rate of lead damages was higher in the TSI group (5.3%) and lower in the PPB group (0.4%). The higher incidence in the TSI group is probably caused by two factors: Firstly, our definition of a lead damage is stricter as insulation defects without impairment of electrical lead parameters were counted as lead damages as well. Secondly, a probable selective underreporting of ‘subclinical’ insulation defects without impairment of electrical lead parameters may translate into a reporting bias that in turn leads to an underestimation of the true incidence of lead damages in the aftermath of generator replacements in the literature.

According to our data, the advantages of the PEAK PlasmaBlade^TM^ are twofold: shortened procedure time and elimination of costly lead replacements due to damage. Because of the large numbers of eligible patients, the implantation of ICDs and pacemakers has the potential to burden national healthcare budgets [14]. Since not all patients eligible receive a device [14], optimization of the surgical procedure associated with lower costs may improve the equity of access to a valuable medical intervention and more patients may benefit from this proven technology. Moreover, Europe has a great regional variation for the utilization of implantable cardiac devices caused by different reasons like reimbursement and guideline adherence but also due to device and implantation related cost [15]. Therefore, the cost savings during device replacement with the use of PEAK PlasmaBlade^TM^ may ease out the financial burden to certain extent and may help to establish the equity in terms of implantation of cardiac devices across different regions. In addition, the shorter occupation of the operating room enables the hospital to use the facility for other interventions leading to overall efficiency in the utilization of healthcare resources.

### Limitations

Our investigation was limited by its retrospective character. Furthermore, we stopped using the conventional strategy after the implementation of the PEAK PlasmaBlade^TM^. As both strategies were never used side by side at either department, a time trend bias cannot be definitively ruled out. The group of patients treated with the conventional strategy outnumbered the PEAK PlasmaBlade^TM^ group by the factor 2. This fact was accounted for by the statistical tests used. The population size of the St. Josef Hospital Braunau was rather small. Nevertheless, for reasons of transparency the data were included into the main analysis and the results presented separately for each hospital.

## Conclusion

Pacemaker and implantable defibrillator generator replacements are associated with a moderate complication risk that cannot be neglected. Our study showed that the use of the PEAK PlasmaBlade^TM^ for generator replacement was efficient, effective and safe. As compared with a conventional strategy applying electrocautery, it was associated with a significantly reduced procedure time while avoiding lead damages. Both these outcomes are likely to translate into considerable cost savings.

## Supporting information

S1 Table Anonymized data set. (XLSX)
